# 

**Published:** 1897-05-22

**Authors:** 


					i'his nufii'ii'AL, A13SULUTIEL.Y PUKE, ttieretore oxudx. 1UA1 V} lOVH
OADBURY'S ww ?*? CADBURY'S
COCOA
1 The standard of highest purity at present
attainable."?Lancet,
isaair Wis tkrn Inlfi&ma&t
No. . Vol. XXII. Saturday
May 22nd, 1897.
[Sahscription^Terms,] pEiICE TWOPENCE.

				

